# The characteristics of cognitive impairment in subjective chronic tinnitus

**DOI:** 10.1002/brb3.918

**Published:** 2018-01-31

**Authors:** Yi Wang, Jian‐Ning Zhang, Wei Hu, Ji‐Jun Li, Jia‐Xuan Zhou, Jian‐Ping Zhang, Guo‐Feng Shi, Ping He, Zai‐Wang Li, Ming Li

**Affiliations:** ^1^ Yunnan University, University of Traditional Chinese Medicine Kunming China; ^2^ Otolaryngological Department Yunnan Province Traditional Chinese Medicine Hospital of Yunnan University of Traditional Chinese Medicine Kunming China; ^3^ Department of Neurology Wuxi People's Hospital of Nanjing Medical University Wuxi China; ^4^ Department of Otolaryngology Yueyang Hospital of Integrated Traditional Chinese and Western Medicine Shanghai China; ^5^ Department of Neurology Taihe Hospital Affiliated to Hubei University of Medicine Shiyan China; ^6^ Department of Integrative Medicine Shanghai Children's Medical Center Shanghai Jiaotong University School of Medicine Shanghai China

**Keywords:** cognitive abilities screening instrument, cognitive impairment, neuropsychology, P300 event‐related potential, subjective chronic tinnitus

## Abstract

**Introduction:**

Subjective chronic tinnitus is a common medical syndrome with a high frequency of cognitive impairment; however, the characteristics of cognitive impairment in chronic tinnitus are poorly understood. Investigating the scope of cognitive impairment across the severity spectrum of tinnitus patients may shed light on the issue.

**Methods:**

A consecutive series of 207 subjective chronic tinnitus patients were classified into mild tinnitus group (*n* = 95) and severe tinnitus group (*n* = 112) by THI score (the cutoff THI scores were 37/38). These patients were assessed using the Cognitive Abilities Screening Instrument (CASI) and P300 event‐related potential.

**Results:**

Although pure tone averages were not different between mild or severe tinnitus patients, severe tinnitus patients scored lower on the CASI assessment as well as almost all subdomains of CASI, particularly in items such as “short‐term memory,” “concentration or mental manipulation,” “orientation,” “abstraction and judgment,” “language abilities,” and “visual construction.” Furthermore, compared to mild tinnitus patients, severe tinnitus patients exhibited longer N2 and P3 latencies. Finally, a correlation analysis revealed that tinnitus severity was negatively correlated with CASI score and positively correlated with N2 and P3 latencies.

**Conclusions:**

This study reveals that tinnitus patients on the severe end of the spectrum may be at risk for serious cognitive deficits, which may not be a secondary response to disease manifestations but a primary feature of the underlying disease.

## 1 INTRODUCTION

Subjective tinnitus is a common medical symptom that describes the conscious perception of an auditory sensation in the absence of a corresponding external stimulus (Baguley, McFerran, & Hall, 2013; Molini, Faralli, Calzolaro, & Ricci, 2014). Tinnitus is not a unitary symptom (Shulman & Goldstein, 2009); rather, evidence is emerging describing that chronic tinnitus patients exhibit cognitive deficits manifesting in the way they handle information (Andersson & McKenna, 2006; Hallam, McKenna, & Shurlock, 2004; Pierce et al., 2012). Despite this, there are still relatively few published studies exploring cognitive function in chronic tinnitus patients (Andersson & McKenna, 2006). Similarly, the etiologies of the cognitive deficits associated with tinnitus remain to be determined. In essence, the characteristics of the cognitive deficits in chronic tinnitus are poorly understood and it remains unclear whether cognitive impairment is a secondary response to disease manifestations or a primary feature of the underlying disease. Consequently, investigating the characteristics of cognitive impairment in subjective chronic tinnitus may shed light on these issues.

Although a recent study has shown that some tinnitus patients exist without obvious cognitive impairments (Waechter & Brannstrom, 2015), many other studies have demonstrated that patients experiencing severe tinnitus may suffer serious cognitive deficits which may lead to an obvious decrease in quality of life and work efficiency (Andersson & McKenna, 2006; Bankstahl & Gortelmeyer, 2013; Das, Wineland, Kallogjeri, & Piccirillo, 2012; Hallam et al., 2004; Pierce et al., 2012). Due to these incongruent research findings and our own clinical observations, we hypothesized that the severity of tinnitus may have a positive relationship with cognitive impairment and, specifically, that partial mild tinnitus patients would have no obvious cognitive deficits while severe tinnitus patients would exhibit more serious cognitive deficits. Moreover, we hypothesized that the cognitive ability of tinnitus patients could be a useful tool for the assessment of tinnitus severity.

In order to study the correlation of tinnitus severity and cognitive impairment in tinnitus patients, we classified tinnitus patients into the mild and severe tinnitus groups by level of severity as ranked according to the THI score developed by Pierce et al. (Newman, Sandridge, & Jacobson, 1998; Pierce et al., 2012). Next, we compared cognitive impairment across tinnitus severity class using the Cognitive Abilities Screening Instrument, Chinese Version 2.0 (CASI C‐2.0; Lin, Wang, Liu, & Teng, 2012; Lin et al., 2002) and P300 event‐related potential (P300 ERPs). Finally, a correlation analysis between severity of cognitive impairment and the extent of cognition deficit was performed. The results of this study may help clinicians to detect and/or improve awareness of the cognitive impairment characteristics of tinnitus patients.

## 2 MATERIALS AND METHODS

### 2.1 Participants

Tinnitus as mentioned in this article refers to subjective chronic tinnitus. The consecutive series of tinnitus patients included in this study were recruited from the Otolaryngological Department at the Yunnan Province Traditional Chinese Medicine Hospital of Yunnan University of Traditional Chinese Medicine and the Yueyang Hospital of Integrated Traditional Chinese and Western Medicine between April 2013 and March 2014. Subjective chronic tinnitus was diagnosed on the basis of criteria previously published by Shulman and Farhadi (Farhadi et al., 2010; Shulman & Goldstein, 2009). All subjects were diagnosed with subjective chronic tinnitus by two fellowship‐trained otolaryngologists. Eligibility criteria included being 18 years to 80 years old and obtaining a diagnosis of definite nonpulsatile subjective chronic tinnitus (unilateral or bilateral for at least 6 months). The cognition of chronic tinnitus patients was assessed using the CASI C‐2.0 score. Dementia was diagnosed according to the Diagnostic and Statistical Manual of Mental Disorders, 4th edition (DSM‐IV) criteria, after a clinical examination that included an interview with a caregiver in addition to cognitive testing. Tinnitus patients were included in this study on condition that they did not suffer from dementia (the cutoff CASI C‐2.0 scores in differentiating between dementia and normal were as follows: 49/50 for Education years = 0; 67/68 for Education years = 1~5; and 79/80 for Education years > 6; Lin et al., 2002). The exclusion criteria also included objective tinnitus and tinnitus caused by abnormal anatomical structure of the external and middle ear. Patients with other neurological or physical diseases with potential influence on hearing capacity were also excluded. Although 403 chronic tinnitus patients were recruited, only 207 chronic tinnitus patients fulfilled these inclusion criteria. The hearing thresholds of all participants were measured using an audiometer (AC40, Interacoustics, Denmark) calibrated in accordance with ISO 389‐1 (Poulsen & Oakley, 2009). Hearing thresholds of 25 dB HL or better at octave intervals 125 to 8,000 Hz were defined as normal hearing (Demeester et al., 2012; Park et al., 2013). The study design was approved by the human ethics committee of Yunnan Institute of Traditional Chinese Medicine. All study participants gave their informed consent.

### 2.2 Measures

Tinnitus patients underwent the Tinnitus Handicap Inventory (THI) (Newman, Jacobson, & Spitzer, 1996) examination to evaluate the severity of their disorder. Tinnitus patients were classified into mild tinnitus group or severe tinnitus group according to THI score (the cutoff THI scores for differentiating severe and mild tinnitus were 37/38; Pierce et al., 2012) as a first step to investigate correlation of tinnitus severity and cognitive impairment in tinnitus patients. The cognitive impairment of tinnitus patients was rated by two fellowship‐trained neurologists using the CASI C‐2.0 scale. The consistent reliability kappa coefficient between the two neurologists was 0.88. The CASI C‐2.0 consists of 20‐item sets which can be divided into nine subdomains, including long‐term memory (LTM), short‐term memory (STM), attention (Att), concentration or mental manipulation (Conc), orientation (Ori), abstraction and judgment (AJ), language abilities (Lang), visual construction (VC), and category fluency (Flu). The CASI C‐2.0 total score ranges from 0 to 100, with a higher score indicating better cognitive ability. The CASI C‐2.0 has been commonly used in dementia research and clinical practice to evaluate a subject's cognitive abilities in China (Lin et al., 2012). In addition, the scale has been carefully developed and its efficacy, including sensitivity and specificity, has been well studied (Tsai, Lin, Wu, & Liu, 2004). Thus, we applied this rating scale to assess cognitive ability in this study.

### 2.3 Event‐related brain potentials

Additionally, P300 ERPs were recorded in all subjects in this study as an objective measure for assessing tinnitus patient cognitive ability. The P300 ERPs is often obtained by a simple discrimination task in which two different stimuli are randomly presented with one occurring less frequently than the other—this is known as the oddball paradigm. Stimulus discrimination is believed to be reflected electrophysiologically as a positive deflection in voltage with a latency of roughly 250–500 ms (Polich, 2007). This paradigm belongs to the ensemble of important tools in electrophysiology which are used as measures of “working memory” (Oniz and Basar, 2009). At the same time, auditory oddball paradigms have the advantage of being simple enough to be performed correctly by most patients. As such, we employed the auditory oddball paradigm in this study.

The P300 wave is an auditory evoked potential which was recorded by Auditory Evoked Potential P300 (Chartr^™^EP, GN otometrics, Denmark) in this study. Assessment of P300 auditory evoked potentials was performed as previously described (Papaliagkas, Kimiskidis, Tsolaki, & Anogianakis, 2008). Briefly, the patients remained quiet and awake while sitting in a comfortable chair. P300 potential recordings were performed in a silent, semidark room. Electrodes were positioned in accordance with the International Electrode System (IES) 10–20 standard. Silver (Ag)/silver chloride (AgCl) electrodes were placed in midfrontal (Fz), midcentral (Cz), and midparietal (Pz) positions. Two reference electrodes were attached to the patients’ earlobes, designated as A1 (representing the left ear) and A2 (representing the right ear). The target tones (2,000 Hz, probability 20%) and nontarget tones (1,000 Hz, probability 80%) were delivered to elicit auditory ERPs (Bailey et al., 2013). The tone intensities were 85‐dB HL for target stimuli and 60‐dB HL for nontarget stimuli, and each stimulus was presented for a duration of 50 ms (including 5‐ms rising and 5‐ms falling time). The stimuli were presented binaurally for 1–3 s in a random order. Subjects were instructed to press a handheld button (response switch) with their dominant hand to indicate target stimulus detection. For each subject, there were 300 trials, and only correctly detected target trials with responses less than 2,000 ms were accepted. The brain activity was recorded at the three sites of active electrodes: Fz, Cz, and Pz as previously described. Data were recorded in the frequency range of 0.1–100 Hz at a 512 Hz sampling rate, with impedances below 5 kΩ. P300 ERPs were recorded using the classical oddball paradigm with target and nontarget tones. When recordings were completed, ERPs were analyzed, and the features of the P300 components were quantified by an experienced technician. Particularly, latencies and amplitudes of N2 and P3 were measured.

### 2.4 Analyses

Differences between the baseline characteristics of two subtinnitus groups were investigated using the Student's *t* test and nonparametric tests. Differences in measured data (age, hearing, pure tone average, and tinnitus duration) were also compared by the Student's *t* test. Nonparametric tests were used for categorical variables such as gender (chi‐square test) and education level (Mann–Whitney test).

To assess whether there was a strong association between hearing loss and cognition, the relationship between pure tone averages (hearing threshold), CASI score (cognitive ability), and P300 ERP parameters (N2 and P3) was calculated by Pearson correlation coefficients. Furthermore, we analyzed the relationships between pure tone averages of both ears (hearing loss) and possible factors impacting hearing (tinnitus severity, tinnitus duration, and age) using Pearson correlation coefficients. Due to nonstatistically significant differences in demographic or clinical characteristics between the mild tinnitus group and severe tinnitus group, *t* tests were used for statistical analysis of P300 ERP parameters (N2 and P3) and CASI C‐2.0 and its subdomains within tinnitus subgroups. Correlation among THI score, CASI C‐2.0 score, and P300 ERP parameters were additionally analyzed using Pearson correlation coefficients. The relationship among CASI C‐2.0 subdomains, THI score, and P300 ERP parameters (N2 and P3) was similarly calculated by Pearson correlation coefficients to assess whether each subdomain was correlated with tinnitus severity or latencies of N2 and P3. All statistical analyses were performed using the Statistical Package for the Social Sciences version 13.0 (SPSS: RRID:SCR_002865, Chicago, IL, USA). All statistical tests were two‐sided and conducted at a significance threshold of *p *=* *.05.

## 3 RESULTS

Of the recruited 403 tinnitus patients, 152 tinnitus patients with neurological or physical diseases potentially influencing hearing were excluded. From the remaining 251 tinnitus patients, 41 patients with a CASI score less than the designated cutoff were excluded as these patients were considered at risk for dementia comorbidity. Finally, 207 patients with tinnitus (95 patients in the mild tinnitus group and 112 patients in the severe tinnitus group) fulfilled the inclusion criteria. Table 1 shows the demographic and clinical characteristics of these patients. There was no significant difference across age, gender, education level, hearing loss (pure tone average of both ears), tinnitus duration, and unilateral/bilateral tinnitus between the two groups.

**Table 1:** Demographic and clinical characteristics of the patients with tinnitus in two groups

	Mild tinnitus group (*n* = 95)	Severe tinnitus group (*n* = 112)	Test statistic	*p* value
Age (yrs: mean ± *SD*)	47.88 ± 12.57	48.18 ± 10.59	*t* = −0.183	.855
Gender (*n*: M/F)	48/47	48/64	*x* ^2^ = 0.927	.336
Education level (*n*: 0 years/1~5 years/>6 years)	19/46/30	12/62/38	*Z *=* *−1.127	.260
Pure tone average of left ear (dB: mean ± *SD*)	31.31 ± 18.57	31.19 ± 18.42	*t* = 0.045	.964
Pure tone average of right ear (dB: mean ± *SD*)	27.26 ± 13.86	27.66 ± 14.20	*t* = −0.202	.840
Tinnitus duration (years: mean ± *SD*)	3.17 ± 5.66	1.98 ± 2.97	*t* = 1.837	.068
Unilateral/bilateral tinnitus (*n*)	32/63	46/66	*x* ^2^ = 0.274	.315

*SD*, standard deviation; M, male; F, female; dB, decibel.

The relationships between cognition (CASI) and tinnitus severity (THI), tinnitus duration, age, and pure tone averages of both ears, which may affect the cognition of patients with tinnitus, were analyzed. Table 2 demonstrates that cognition had a negative relationship with tinnitus severity (THI), age, and pure tone averages of both ears but not tinnitus duration. In order to analyze the correlation between hearing loss and cognition, the relationship between pure tone average (hearing threshold), CASI score (cognitive ability), the parameters of P300 ERPs (latencies of N2 and P3) was calculated by Pearson correlation coefficients. The results revealed that the hearing threshold of both ears had a negative correlation with CASI score (*r* = −.138 and *r* = −.143, respectively, both *p* values < .05) and a positive correlation with the latency of P300 (*r* = .176 and *r* = .211, respectively, both *p* values < .05) and latency of N2 (*r* = .143 and *r* = .206, respectively, both *p* values < .05). This suggested that there was a strong association between hearing loss and cognition. Considering that hearing loss exhibited a close relationship with cognition in tinnitus patients, we further analyzed the relationships between hearing threshold (pure tone averages of both ears) and possible factors impacting hearing (tinnitus severity, tinnitus duration, and age). The result showed that only age but not tinnitus severity or tinnitus duration of the patients was positively correlated with hearing loss (Table 3).

**Table 2:** The relationships between cognition (CASI) and possible factor impacting cognitive ability (tinnitus severity, tinnitus duration age, and pure tone averages of both ears)

Statistical value	THI	Tinnitus duration	Age	Pure tone averages of right ear	Pure tone averages of left ear
*r* value	−.865	.130	−.160	−.138	−.143
*p* value	<.001	.062	.021	.047	.039

**Table 3:** The relationships between hearing threshold (pure tone averages of both ears) and possible factor impacting hearing (tinnitus severity, tinnitus duration, and age)

Hearing threshold	Statistics value	THI	Tinnitus duration	Age
Pure tone average of left ear	*r* value	−.020	−.040	.222
	*p* value	.776	.564	.001
Pure tone average of right ear	*r* value	−.039	.072	.283
	*p* value	.578	.301	<.001

The CASI total score and the mean scores of the CASI subdomains of the two tinnitus subgroups are presented in Table 4. The cognitive deficits detected within tinnitus groups (mild tinnitus patients and severity tinnitus patients) were further analyzed to investigate the correlation between tinnitus severity and the extent of cognitive impairment. The results revealed that patients with severe tinnitus exhibited a lower score in almost all CASI subdomains (except “long‐term memory”) compared to the mild tinnitus group (all *p* < .05). Particularly, the performance of severe tinnitus patients was worse than the mild tinnitus group across the following subdomains: “short‐term memory,” “concentration or mental manipulation,” “orientation,” “abstraction and judgment,” “language abilities,” and “visual construction” (all *p *<* *.001). These results were observed in the absence of significant differences in hearing loss between the two groups (Table 4).

**Table 4:** Distribution of CASI subdomain scores and CASI sum score in two tinnitus groups

CASI subdomains and sum score	Mild tinnitus group Mean ± *SD*	Severe tinnitus group Mean ± *SD*	*t* value	*p* value
LTM	8.72 ± 1.30	8.45 ± 1.38	1.436	.153
STM	8.15 ± 1.99	5.67 ± 1.74	9.548	<.001
Att	7.74 ± 0. 69	7.46 ± 0.76	2.689	<.008
Conc	9.42 ± 0.92	8.38 ± 1.62	5.537	<.001
Ori	8.03 ± 0.89	6.51 ± 1.41	9.102	<.001
AJ	10.13 ± 1.45	8.21 ± 1.70	8.629	<.001
Lang	9.65 ± 0.71	8.96 ± 1.10	5.318	<.001
VC	8.99 ± 1.12	7.96 ± 1.57	5.377	<.001
Flu	7.68 ± 1.58	7.13 ± 1.88	2.297	.023
Sum	87.04 ± 7.08	73.98 ± 8.64	11.759	<.001

No significant difference in demographic or clinical characteristics was detected between mild tinnitus group and severe tinnitus group; *t* tests were used for statistical analysis. CASI, Cognitive Abilities Screening Instrument; *SD*, standard deviation; LTM, long‐term memory; STM, short‐term memory; Att, attention; Conc, concentration or mental manipulation; Ori, orientation; AJ, abstraction and judgment; Lang, language abilities; VC, visual construction; Flu, category fluency.

In order to objectively investigate tinnitus patient cognitive ability, P300 ERPs were recorded from all subjects in this study. The latencies and amplitudes of N2 and P3 waveforms are shown for the two groups in Table 5. Compared to mild tinnitus patients, severe tinnitus patients exhibited longer P300 (*p *<* *.001) and N2 (*p* < .05) latencies though there was no significant difference in P300 or N2 amplitude (Table 5).

**Table 5:** The P300 event‐related potential parameters in two tinnitus groups

P300 ERP parameters		Mild tinnitus group Mean ± *SD*	Severe tinnitus group Mean ± *SD*	*t* value	*p* value
N2	Latency (ms)	232.17 ± 34.75	241.02 ± 26.48	−2.032	.044
	Amplitude (μV)	−3.36 ± 2.21	−3.29 ± 2.46	−0.227	.821
P3	Latency (ms)	307.87 ± 46.33	345.88 ± 40.72	−6.282	<.001
	Amplitude (μV)	10.89 ± 7.95	10.54 ± 8.58	0.299	.765

An analysis of covariance was used for statistical analysis using hearing threshold (pure tone average of two ears) as a covariate. ERP, event‐related potential; *SD*, standard deviation; ms, milliseconds; μV, microvolts.

**Figure 1:**
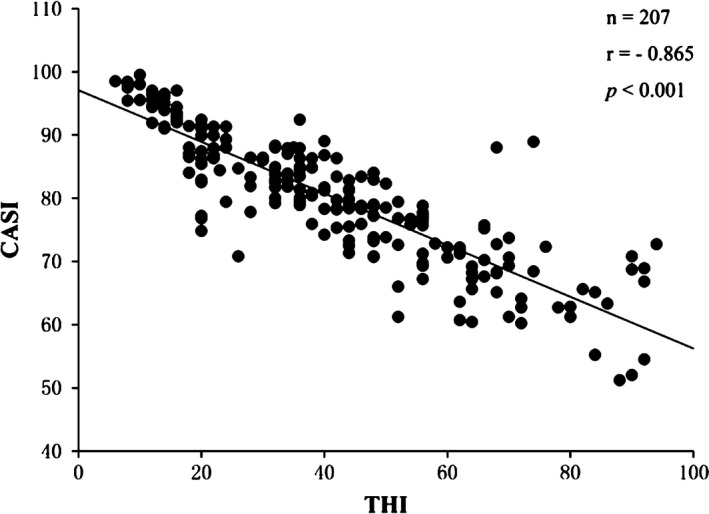
Correlation analysis scatterplot between Cognitive Abilities Screening Instrument (CASI) scores and Tinnitus Handicap Inventory (THI) scores showing a negative correlation between the two scores (*n* = 207, *r* = −.865, *p *<* *.001)

**Figure 2:**
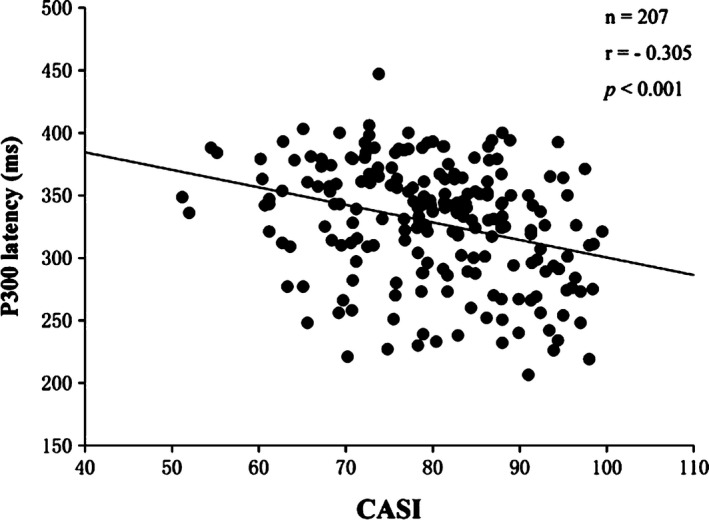
Correlation analysis scatterplot between Cognitive Abilities Screening Instrument (CASI) scores and P3 latency showing a negative correlation between the two items (*n* = 207, *r* = −.305, *p *<* *.001)

**Figure 3:**
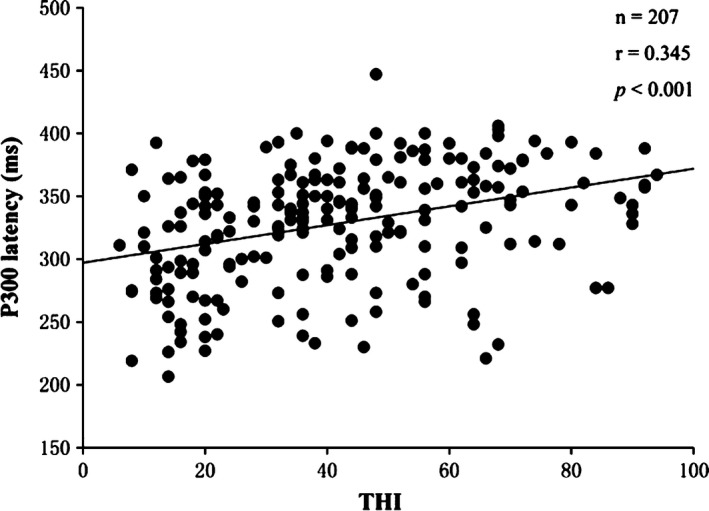
Correlation analysis scatterplot between Tinnitus Handicap Inventory (THI) scores and P3 latency showing a positive correlation between the two items (*n* = 207, *r* = .345, *p *<* *.001)

To analyze the correlation between tinnitus severity and cognitive ability, the relationship between THI score, CASI score, and P300 ERP parameters was calculated using Pearson correlation coefficients. The correlation analysis revealed that CASI score was negatively correlated with THI score (*r* = −.865, *p *<* *.001), P3 latency (*r* = −.305, *p *<* *.001), and N2 latency (*r* = −.208, *p* = .003). Additionally, the results showed that the CASI score had no correlation with the P300 amplitude (*r* = −.010, *p *=* *.884) or the N2 amplitude (*r* = .023, *p* = .741). Figure 1 shows the correlation between the CASI score and THI score while Figure 2 shows the correlation between the CASI score and the latency of P300 in the two groups. The latencies of N2 and P3 were both positively correlated with THI score (*r* = .345, *p *<* *.001; *r* = .249, *p* < .001) while the N2 and P3 amplitudes were not correlated with THI score (*r* = .345, *p *=* *.668; *r* = −.022, *p* = .755). Finally, Figure 3 presents the correlation between latency of P300 and THI score.

To assess whether tinnitus severity was correlated with CASI subdomains, subdomain scores were independently analyzed with THI scores using Pearson correlation coefficients. The correlation analysis revealed that there was a negative correlation between each CASI subdomain score and THI score in the tinnitus experimental groups (Pearson coefficient *r* range −.752 to −.297, all *p* values < .001, in Table 6). Finally, to investigate the correlation between CASI subdomains and N2 and P3 latencies, the relationship between all CASI subdomains and the latencies of N2 and P3 was calculated by Pearson correlation coefficients. Table 7 shows that almost all of the CASI subdomains (except “long‐term memory”) were negatively correlated with P3 latency. Table 7 also shows that only three CASI subdomains (“short‐term memory,” “orientation,” and “abstraction and judgment”) were negatively correlated with N2 latency.

**Table 6:** The relationships between CASI subdomains and tinnitus severity (THI)

CASI subdomains	Score (Mean ± *SD*)	*r* value	*p* value
LTM	8.57 ± 1.35	−.615	<.001
STM	6.81 ± 2.23	−.752	<.001
Att	7.59 ± 0.74	−.297	<.001
Conc	8.86 ± 1.44	−.519	<.001
Ori	7.21 ± 1.42	−.539	<.001
AJ	9.09 ± 1.85	−.648	<.001
Lang	9.28 ± 1.00	−.471	<.001
VC	8.43 ± 1.47	−.610	<.001
Flu	7.38 ± 1.76	−.657	<.001

CASI, Cognitive Abilities Screening Instrument; *SD*, standard deviation; LTM, long‐term memory; STM, short‐term memory; Att, attention; Conc, concentration or mental manipulation; Ori, orientation; AJ, abstraction and judgment; Lang, language abilities; VC, visual construction; Flu, category fluency.

**Table 7:** The relationships between CASI subdomains and the latencies of N2 and P3

CASI subdomains	Score (Mean ± *SD*)	N2		P3	
		*r* value	*p* value	*r* value	*p* value
LTM	8.57 ± 1.35	.037	.600	.006	.897
STM	6.81 ± 2.23	−.197	.005	−.246	<.001
Att	7.59 ± 0.74	−.075	.282	−.114	.020
Conc	8.86 ± 1.44	−.104	.136	−.173	<.001
Ori	7.21 ± 1.42	−.261	<.001	−.340	<.001
AJ	9.09 ± 1.85	−.140	.045	−.132	<.001
Lang	9.28 ± 1.00	−.044	.527	−.691	<.001
VC	8.43 ± 1.47	−.110	.113	−.192	<.001
Flu	7.38 ± 1.76	−.040	.569	−.605	<.001

CASI, Cognitive Abilities Screening Instrument; *SD*, standard deviation; LTM, long‐term memory; STM, short‐term memory; Att, attention; Conc, concentration or mental manipulation; Ori, orientation; AJ, abstraction and judgment; Lang, language abilities; VC, visual construction; Flu, category fluency.

## 4 DISCUSSION

Cognitive impairment is an inclusive term used to describe any characteristic that acts as a barrier to the cognitive process (Auchter, Williams, Barksdale, Monfils, & Gonzalez‐Lima, 2014), and this describes not only deficits in global intellectual performance but also specific deficits in cognitive abilities (dysmnesia, learning disorders, etc.)(Bralet, Navarre, Eskenazi, Lucas‐Ross, & Falissard, 2008; Wegener, Marx, & Zettl, 2013). Cognitive impairment may have a close relationship with tinnitus, as tinnitus is not only an aberrant auditory sensory perception but also associated with a variety of nonauditory symptoms which include frustration, inability to relax, and difficulty concentrating (Wineland, Burton, & Piccirillo, 2012). Clinicians have long been aware of the high frequency of cognitive impairment in tinnitus patients, yet little is known about the characteristics of the cognitive impairment in tinnitus and the etiology of cognitive impairment that may be associated with tinnitus remains unclear. Assessment of cognitive impairment in tinnitus has a potentially important clinical significance and, if substantiated, could emerge as an important clinical feature of tinnitus patients. Consequently, the role of cognitive impairment in tinnitus should be explored.

Current data on the characteristics of cognitive deficits in tinnitus patients are limited. Some studies have documented cognitive deficits in tinnitus patients including inattention, instant memory disorders, and inefficient learning as the most common symptoms (Das et al., 2012; Hallam et al., 2004). However, a recent study has actually concluded that tinnitus patients did not exhibit any obvious cognitive impairment (Waechter & Brannstrom, 2015). The inconsistency across reported findings may due to the differences in the empirical approaches adopted (e.g., size of sample or cognition rating scale) in those studies. Additionally, cognitive impairment may be differentially manifested depending on the severity of the tinnitus cohorts used in each study. Accordingly, we hypothesized that severity of tinnitus patients may be positively correlated with extent of cognitive impairment and consequently that severe tinnitus patients could have more conspicuous cognitive deficits than mild tinnitus patients. Several reports have documented the elevation of cognitive deficits in patients with tinnitus as well as correlated the severity of these symptoms with tinnitus severity (Andersson & McKenna, 2006; Bankstahl & Gortelmeyer, 2013; Das et al., 2012; Hallam et al., 2004; Pierce et al., 2012). Although these studies have reported the symptomatic profile of the cognitive deficits in patients with tinnitus, to our knowledge, no previous study has systematically investigated the characteristics of cognitive deficits in tinnitus patients and analyzed the correlation between tinnitus severity and cognitive impairment using combinatorial subjective and objective methods.

In order to compare the cognitive abilities between two tinnitus patient subgroups and investigate the correlation between tinnitus severity and severity of cognitive impairment, we used the Cognitive Abilities Screening Instrument (CASI) and P300 ERPs to assess cognitive ability. The results revealed that patients with severe tinnitus had a lower total score on the CASI score as well as almost all subdomains of CASI (except “long‐term memory”) than mild tinnitus patients even without significant difference in hearing loss between the two groups in this study. Particularly, the performance of severe tinnitus patients was obviously worse than the mild tinnitus groups in the subdomains “short‐term memory,” “concentration or mental manipulation,” “orientation,” “abstraction and judgment,” “language abilities,” and “visual construction” (all *p *<* *.001). These results suggested that cognitive impairment of severe tinnitus patients was greater than in mild tinnitus patients, implying that extent of cognitive impairment may be closely associated with the severity of tinnitus.

It is typically insufficient to assess cognitive functions using neuropsychological tests, due to the presence of many confounding variables which may affect cognitive functions. The P300 ERPs is a reflection of cognitive processing. Objective measures like P300 ERPs may thus be appropriate to supplement studies involving cognitive performance assessments. N2 and P3 latencies are related to stimulus evaluation processes such as encoding and classification (Giroud, Lemke, Reich, Matthes, & Meyer, 2017; Ozdemir, Demiroren, Demir, & Serin, 2014; Santos Filha & Matas, 2010). In this study, there was a significant difference in N2 and P3 latencies between the two tinnitus subgroups, further illustrating that cognitive deficits might be characteristic of tinnitus. Although N2 and P3 amplitudes also reflect the effort of attention allocation (Novak & Foti, 2015; Ozdemir et al., 2014), there was no significant difference in the N2 and P3 amplitudes detected between the two patient subgroups in this study. Instead, our results described that the N2 and P3 amplitudes did not correlate with THI score or CASI scores, indicating that N2 and P3 amplitudes were likely not sensitive to cognitive dysfunction manifested by patients in this study.

The correlation analysis indicated that almost all of the CASI subdomains (except “long‐term memory”) have negative correlations with P3 latency, which indicated that both CASI scale and P3 latency were effective methods for the detection of tinnitus‐related cognitive dysfunction. N2 latency may also be an effective method for assessment of tinnitus‐related cognitive ability, although only three CASI subdomains (“short‐term memory,” “orientation,” and “abstraction and judgment”) showed negative correlation with N2 latency. Taken together, the subdomains “short‐term memory” and “orientation” appeared to be the two most relevant subdomains as their combined score was shown to be more effective than the total score in assessing cognitive ability (Tsai et al., 2004). Meanwhile, the correlation analysis revealed that there was a negative relationship between each CASI subdomain score and THI score in the tinnitus experimental groups and the CASI score was also negatively correlated with THI score. The results suggested that tinnitus severity was highly correlated with the severity of cognitive impairment of tinnitus patients. Furthermore, not only P3 latency but also N2 latency was found to be positively correlated with THI score (tinnitus severity). Altogether, the correlation analysis results demonstrated that severity of tinnitus might have a positive correlation with the extent of cognitive impairment, and in other words, severe tinnitus patients may be more prone to serious cognitive deficits compared to mild tinnitus patients.

Although there were no tinnitus patients comorbid for dementia in this study, the tinnitus patients did experience obvious cognitive impairments. It remains unclear why these cognitive deficits appear so severely in tinnitus patients, especially in severe tinnitus patients. Several recent tinnitus studies may provide some clues to explain this phenomenon. Some studies have reported potentially important structural differences in the brains of people with tinnitus. Leaver et al. confirmed that people with chronic tinnitus exhibit reduced gray matter in the ventromedial prefrontal cortex (vmPFC) compared to controls matched for age and hearing loss (Leaver et al., 2012). Ventromedial prefrontal cortex (vmPFC) damage can subsequently impair the cognitive components of naturalistic event comprehension and memory (Zacks, Kurby, Landazabal, Krueger, & Grafman, 2016). A study by Lee et al. (2007), employing diffusion tensor imaging techniques, demonstrated that the arcuate fasciculus connecting the auditory and frontal regions was damaged in the brain of tinnitus patients. The study suggested a deterioration of white matter fibers in people with tinnitus and concluded that cortical interconnectivity is an important component of recognition ability (Lee et al., 2007; Viirre, 2007). Compromised recognition ability in turn may be mediated by abnormal arcuate fasciculus‐related reading and visuospatial deficits (Rauschecker et al., 2009). Moreover, Vanneste et al. demonstrated that cognitive changes in tinnitus patients are associated with changes in hippocampal activity as well as alteration in the anterior cingulate and insula (Vanneste, Faber, Langguth, & De Ridder, 2016). In short, these structural anomalies in the brain of tinnitus patients may constitute the intrinsic features of cognitive impairment. Further, these observations suggest that tinnitus‐related cognitive impairment is not a secondary response to the manifestation of the disease. Collectively, the aforementioned studies’ and our current findings support cognitive impairment as a primary feature of tinnitus.

The present study has some limitations. First, tinnitus patients may suffer from both tinnitus‐related distress and depression, two distinct emotional states (Joos, Vanneste, & De Ridder, 2012). It may be possible that the severity and frequency of tinnitus‐related emotional states (distress and depression) in the two tinnitus groups are significantly different. New insights into the neurobiology of tinnitus suggest that neuronal changes occur not only on the classical auditory pathways but also on the insula, anterior cingulate cortex (ACC), amygdala, dorsolateral prefrontal cortex, orbitofrontal cortex, and (para) hippocampus (PHC; Golm, Schmidt‐Samoa, Dechent, & Kroner‐Herwig, 2016; Joos et al., 2012; Simonetti & Oiticica, 2015). These neuroanatomical changes play a specific role in both tinnitus‐related emotional state disorder and cognitive deficits (Araneda et al., 2015; Kraus & Canlon, 2012; Simonetti & Oiticica, 2015; Vanneste et al., 2016). Thus, tinnitus‐related emotional state disorder (distress and depression) has exhibited a close relationship with tinnitus and cognitive deficits (Ghodratitoostani et al., 2016; Tegg‐Quinn, Bennett, Eikelboom, & Baguley, 2016). At the same time, patients with poor cognitive skills may be more likely to harbor misconception about tinnitus, which in turn may lead to clinical symptoms such as fear, anxiety, insomnia, and other harmful psychological reactions. These psychological reactions in turn may deteriorate tinnitus symptoms, forming a cycle of negative outcomes which may interfere with or exacerbate intrinsic cognitive impairments. Although it is still unclear which factors initiate the cycle of tinnitus‐related cognitive impairment, the differential emotional states (distress and depression) between the two groups may introduce a bias in the assessment of cognitive ability. Second, the subjects were all recruited from the Traditional Chinese Medicine Hospital of Yunnan Province and the Yueyang Hospital of Integrated Traditional Chinese and Western Medicine, which may not be representative of the general population. As the prevalence of tinnitus in hospital samples is typically different from that in a community sample, the samples in this study may have introduced another bias. Additionally, the mixed application of parametric and nonparametric tests used to analyze the differences between the baseline characteristics of the two tinnitus subgroups may also lead to bias. Finally, the independent component analysis (ICA), a blind source separation (BSS) technique that is completely data‐driven (Vanneste, Congedo, & De Ridder, 2014), was not applied to data in this study. Thus, there may exist, to a certain extent, a bias introduced by the investigators’ subjective predictions on the relationship between tinnitus severity and cognitive impairments.

In conclusion, in this study, we analyzed the differences in cognitive deficits between mild tinnitus patients and severe tinnitus patients using a combinatorial subjective (CASI) and objective (P300 ERPs) approach. Further, we studied the correlation between the severity of tinnitus and cognition impairment extent. Our findings demonstrated that severe tinnitus patients possess distinct cognitive deficits compared to mild tinnitus patients, a difference that could not be accounted for by differences in hearing loss. Furthermore, the correlation analysis indicated that severity of tinnitus was positively correlated with the extent of cognitive impairment. These data demonstrate that cognitive impairment symptoms may not be a secondary response to disease manifestations but a primary feature of the underlying disease. These findings may also improve clinician awareness of the characteristics of cognitive impairment in tinnitus, which may contribute to improved diagnosis and treatment for tinnitus patients.

## CONFLICT OF INTERESTS

The authors declare no conflict of interests, financial or otherwise.
